# P-1314. Activity of aztreonam-avibactam against Enterobacterales isolated from patients with intra-abdominal infection from Europe, Asia, and Latin America (2020-2024)

**DOI:** 10.1093/ofid/ofaf695.1502

**Published:** 2026-01-11

**Authors:** Helio SaderRodrigo E Mendes, Katherine Perez, Gregory Stone, Marisa Winkler, Mariana Castanheira

**Affiliations:** Element Iowa City (JMI Laboratories), North Liberty, IA; Pfizer, Inc., Groton, Connecticut; Pfizer, Inc., Groton, Connecticut; Element Materials Technology/Jones Microbiology Institute, North Liberty, Iowa; Element, North Liberty, IA

## Abstract

**Background:**

Aztreonam-avibactam (ATM-AVI) has recently been approved for clinical use in the European Union and United States to treat Gram-negative infections, including those caused by metallo-β-lactamase (MBL) producers. We evaluated the activity of ATM-AVI against Enterobacterales (ENT) isolated from patients with intra-abdominal infections (IAI).

**Figure:**
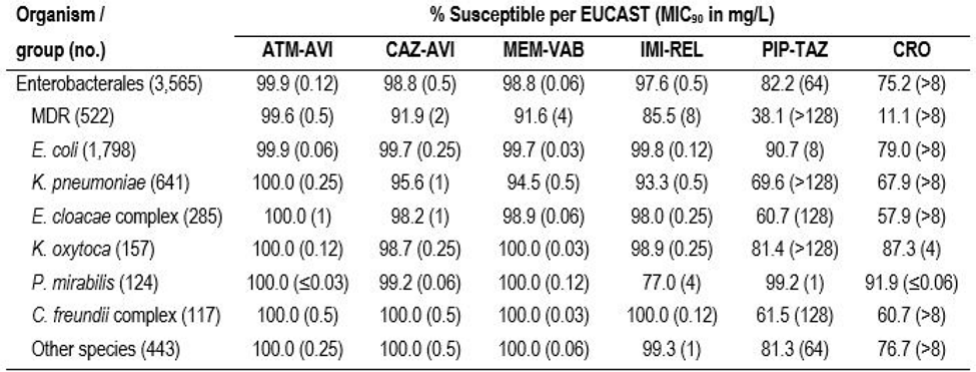
Activity of β-lactamase inhibitor combinations and ceftriaxone against Enterobacterales from intra-abdominal infections Abbreviations: ATM-AVI, aztreonam-avibactam; CAZ-AVI, ceftazidime-avibactam; MEM-VAB, meropenem-vaborbactam; IMI-REL, imipenem-relebactam; PIP-TAZ, piperacillin-tazobactam; CRO, ceftriaxone; MDR, multidrug-resistant.

**Figure:**
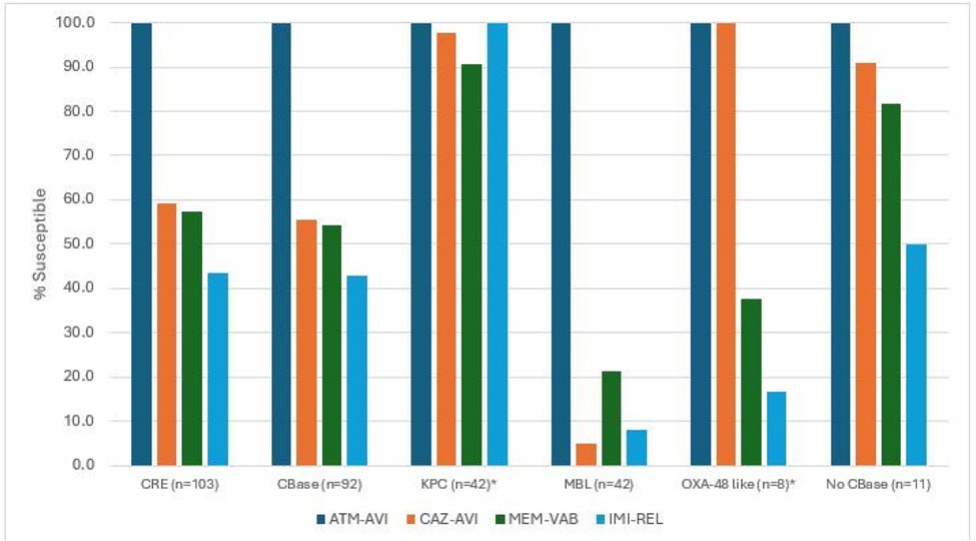
Activity of selected β-lactamase inhibitor combinations against carbapenem-resistant Enterobacterales (CRE) stratified by carbapenemase (CBase) type * Exclude MBL co-producers.

**Methods:**

3,565 isolates (1/patient) were consecutively collected from patients with IAI in 52 medical centers from 28 countries located in Europe, Asia-Pacific region, and Latin America in 2020-2024. Isolates were susceptibility tested by broth microdilution. Carbapenem-resistant ENT (CRE) were screened for carbapenemases (CBase) by whole genome sequencing.

**Figure:**
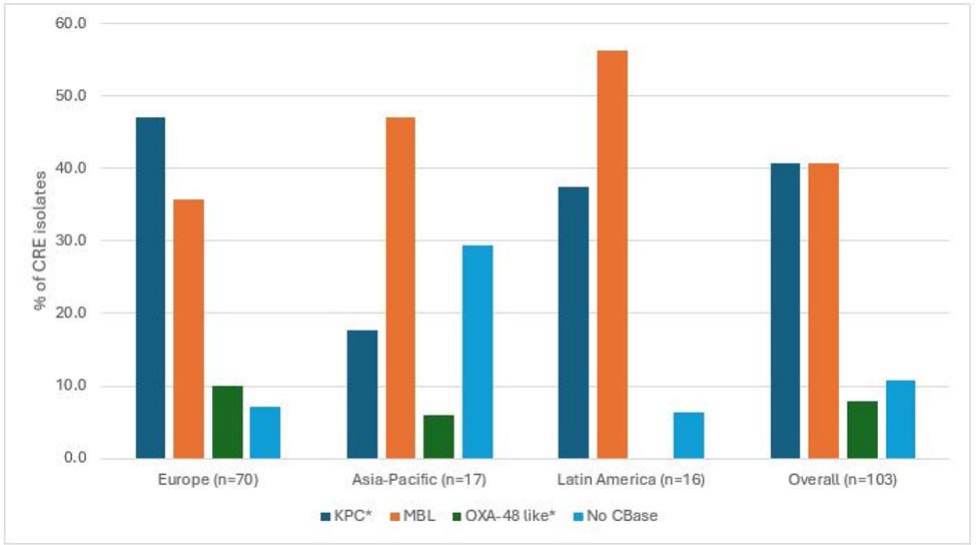
Distribution of carbapenemase (CBase) types by region * Exclude MBL co-producers.

**Results:**

The most common ENT species were *E. coli* (50.4%), *K. pneumoniae* (18.0%), and *E. cloacae* species complex (8.0%). Susceptibility by species is shown in Table 1. Only 2 ENT exhibited ATM-AVI MIC > 4 mg/L (MIC_50/90_, ≤0.03/0.12 mg/L), 2 *E. coli* with ATM-AVI MIC of 8 mg/L. A multidrug-resistant (MDR) phenotype (not susceptible [S] to ≥ 3 classes per EUCAST) was observed in 14.6% of ENT (*n*=522) and the most active β-lactamase inhibitor combination (BLIC) against MDR isolates were ATM-AVI (99.6% S), CAZ-AVI (91.9% S), and MEM-VAB (91.6% S). Piperacillin-tazobactam (PIP-TAZ) was active against 82.2% of ENT and 38.1% of MDR, and meropenem was active against 97.4% of ENT and 82.2% of MDR. Only ATM-AVI showed good activity against CRE (MIC_50/90_, 0.25/1 mg/L, 100.0% S); CAZ-AVI, MEM-VAB, and IMI-REL were active against 59.2%, 57.3%, and 43.4% of CRE, respectively (Figure 1). A CBase was identified in 92 isolates (89.3% of CREs), and included KPCs (44.7% of CREs), MBLs (40.8%), and OXA-48 type (15.5%); 11.7% of CRE had 2 CBase types. All CBase producers were ATM-AVI-S whereas susceptibility to CAZ-AVI, MEM-VAB, and IMI-REL were 55.4%, 54.3%, and 42.9%, respectively. The frequencies of CBases varied markedly by region (Figure 2).

**Conclusion:**

ATM-AVI exhibited almost complete activity against ENT causing IAI in EU, Asia, and LA hospitals. The activities of other BLICs against CRE were compromised by the high occurrence of MBL and OXA-48-like producers.

**Disclosures:**

Helio Sader, United States Food and Drug Administration: FDA Contract Number: 75F40123C00140 Rodrigo E. Mendes, PhD, GSK: Grant/Research Support|Shionogi & Co., Ltd.: Grant/Research Support|United States Food and Drug Administration: FDA Contract Number: 75F40123C00140 Katherine Perez, PhD, Pfizer: Stocks/Bonds (Public Company) Marisa Winkler, MD, PhD, Basilea: Advisor/Consultant|Basilea: Grant/Research Support|GSK: Advisor/Consultant|GSK: Grant/Research Support|Melinta Therapeutics: Advisor/Consultant|Melinta Therapeutics: Grant/Research Support|Mundipharma: Advisor/Consultant|Mundipharma: Grant/Research Support|Pfizer: Advisor/Consultant|Pfizer: Grant/Research Support|Pulmocide: Advisor/Consultant|Pulmocide: Grant/Research Support Mariana Castanheira, PhD, Melinta Therapeutics: Advisor/Consultant|Melinta Therapeutics: Grant/Research Support

